# Potential of Silicon Amendment for Improved Wheat Production

**DOI:** 10.3390/plants7020026

**Published:** 2018-03-28

**Authors:** Olga S. Walsh, Sanaz Shafian, Jordan R. McClintick-Chess, Kelli M. Belmont, Steven M. Blanscet

**Affiliations:** 1 Department of Plant Sciences, Southwest Research and Extension Center, University of Idaho, Parma, ID 83660, USA; sanazs@uidaho.edu (S.S.); jrmcclintick@uidaho.edu (J.R.M.-C.); 2 Seminis, Payette, ID 83661, USA; kelli.belmont@gmail.com; 3 BASF-Chemical Co., Caldwell, ID 83605, USA; idahoparma83660@gmail.com

**Keywords:** wheat, silicon, grain yield, protein content

## Abstract

Many studies throughout the world have shown positive responses of various crops to silicon (Si) application in terms of plant health, nutrient uptake, yield, and quality. Although not considered an essential element for plant growth, Si has been recently recognized as a “beneficial substance” or “quasi-essential” due to its important role in plant nutrition, especially notable under stressed conditions. The goal of this study was to evaluate the effect of Si on wheat plant height, grain yield (GY), and grain protein content (GP). The experiment was conducted during two consecutive growing seasons in Idaho. A split-plot experimental design was used with three Si fertilization rates (140, 280, and 560 kg Si ha^−1^) corresponding to 100, 50, and 25% of manufacturer-recommended rates and two application times—at planting and tillering (Feekes 5). MontanaGrow^TM^ (0-0-5) by MontanaGrow Inc. (Bonner, MT, USA) used in this study is a Si product sourced from a high-energy amorphous (non-crystalized) volcanic tuff. There was no significant effect of Si rate and application time on plant height, nutrient uptake, GY, or GP of irrigated winter wheat grown in non-stressed conditions. These results could be directly related to the Si fertilizer source used in the study. We are planning to further evaluate Si’s effect on growth and grain production of wheat grown in non-stressed vs. stressed conditions utilizing several different Si sources and application methods.

## 1. Introduction

Wheat (*Triticum aestivum*, L.) is a major global commodity, both in terms of cultivated area and tradeable value, and is a staple in household diets. Along with rice (*Oryza sativa*, L.) and maize (*Zea mays*, L.), wheat is the one of the most abundant and cultivated crops on Earth, with annual production of approximately 2.7 billion tons [1]. In terms of food security, wheat is an important source of nourishment, providing 30% of calories and 60% of protein consumed by people worldwide [2]. World demand for wheat is rising rapidly as the global population continues to increase. World Bank estimates the demand for wheat in developing countries will increase 60% by 2050 [3]. Wheat growers around the world need to increase their productivity, while the prices of wheat and other cereal grains decrease. In addition, with the expectation that prices for fertilizers and chemicals will continue to rise in the future, wheat producers must substantially improve their production efficiency to stay competitive. Grain yield (GY) and quality are the most important parameters affecting gross returns for wheat producers. In addition, wheat needs to reach specific quality criteria to be eligible for export [4]. 

Balanced mineral nutrition is the key to high-yielding and high-quality wheat production. Crop nutrition studies have recently shown that micronutrients are as important as macronutrients for crop growth and development [5]. Even though silicon (Si) was listed among the elements required for plant life as early as 1912 [6], its importance for higher plants’ growth and development is still debatable due to the lack of evidence demonstrating Si’s direct role in plant metabolism [7,8]. Although Si is not currently recognized as an essential plant nutrient, it has recently been classified as a “beneficial substance” or “quasi-essential” element by the Association of American Plant Food Control Officials (AAPFCO), based on growing evidence of its functionality for a number of crops [9,10,11,12]. Rafi et al. [13] found that Si deprivation causes physical abnormalities in wheat in terms of plant growth, development, and reproduction. In rice, Si-limiting conditions during the reproductive stage resulted in decreases in the dry weights of straw (stem + leaf blade) and grain by 20 and 50%, respectively, compared with plants grown in conditions with adequate Si levels throughout the growing season [14].

Silicon is the second most abundant element in the earth’s crust, but it is present in a polymerized form and is not readily available for plant uptake. Long-term intensive crop cultivation has also been found to result in depletion of plant-available Si from the soils [15]. Plants can only absorb the depolymerized soluble form of Si—the mono silicic acid (H_2_SiO_4_). This soluble form of Si is easily taken up by plant roots and accumulated in plant tissues, with typical concentrations ranging from 0.1 to 10% [10]. Grasses and cereals such as wheat typically contain 1 to 3% Si [9] and fall within a group of plants considered to be high Si accumulators, containing 10 to 100 g Si kg^−1^ dry weight [7,16]. Tubana et al. [11] reported the estimated shoot Si uptake for wheat to be approximately 108 kg Si ha^−1^, which is in agreement with the findings by Heckman [17], who measured Si uptake in wheat straw between 73 kg Si ha^−1^ and 133 kg Si ha^−1^, depending on the Si source applied. The accumulated Si is deposited within the leaf epidermis, where it becomes condensed into a polymerized silica gel (SiO_2_·nH_2_O) known as a phytolith [18]. Phytoliths are immobile and make up a protective structural layer in plant cell walls [15], which acts as an anti-stress factor and helps to alleviate stresses in a wide variety of plant species [19,20,21].

The benefits of Si to agricultural crops growing under stressed conditions have been investigated in several studies. Janislampi [22] observed that, under conditions of drought and salt stress, wheat biomass at vegetative growth stages increased 17% due to Si application. In another study, Balakhnina et al. [23] found that application of Si enhanced the growth of barley (*Hordeum vulgare*, L.) roots and shoots under optimal watering and decreased the intensity of oxidative destruction under soil flooding without significant changes in the activities of antioxidant enzymes. This is an important finding for cereals grown in areas in which intermittent flooding due to standing water in the fields is probable following excessive precipitation. In a different study, Ahmad et al. [5] showed that Si application to wheat grown under drought stress increased K^+^ concentration in the shoots (28.65 mg g^−1^) and grain (3.51 mg g^−1^), which helped to maintain water potential within the wheat plants, ultimately enhancing biomass production and GY. Recently, Saleh et al. [24] found that, in wheat grown under salinity stress, Si application increased shoot dry weight, chlorophyll content, catalase (CAT) activity, glycine betaine (GB) concentration, and superoxide dismutase (SOD) enzyme activity. 

In other studies, scientists have focused on assessing the effect of rate, source, and time of Si application to plants grown under normal (non-stressed) conditions. Singh et al. [25] conducted a study to determine the effect of rate and time of Si application on Si uptake, growth, and yield of rice. They applied three rates of Si (60, 120, 180 kg ha^−1^) at five times throughout the growing season. Their results showed that increasing application rates of Si up to 120 kg Si ha^−1^ significantly increased dry matter, flag leaf effectivity, yield attributes, and yield of rice, compared to the 60 kg Si ha^−1^ rate. They found that rice grain test weight improved when Si was applied at the 180 kg Si ha^−1^ rate. Rice Si content and uptake increased throughout the length of the growing season all the way until harvest, as reflected by the increased uptake and Si content. Application of all Si to the soil prior to planting was superior to other time of Si application, because it was more cost effective and resulted in increased rice growth, yield attributes, yield, and nutrient uptake. 

Marodin et al. [26] evaluated the phyto technical characteristics and the productivity of tomato (*Solanum lycopersicum*, L.) plants under varying rates and sources of applied Si. They assessed Si sources (calcium silicate—Ca_2_SiO_4_, potassium silicate—K_2_SiO_3_, and sodium silicate—Na_2_SiO_3_) at five rates (equivalent to 0, 100, 200, 400, and 800 kg SiO_2_ ha^−1^) applied to tomatoes. Their results showed that fertilization with Si increased commercial productivity of tomato plants. In addition, they showed that calcium and potassium silicates linearly increased Si levels in tomato leaves with increased Si application rates. On the other hand, Si content of tomato leaves decreased at higher Na_2_SiO_3_ application rates.

Jafari et al. [27] evaluated the effect of various rates and application times of Si foliar spray on yield and yield components of beans (*Phaseolus vulgaris*, L.). They showed that foliar Si spray at stem elongation and beginning of flowering significantly increased bean GY by 38% and 21%, respectively, compared to the no-Si control. They found that bean GY was increased mainly due to increase in number of seeds per plant and higher 100-seeds weight. In another study, White et al. [28] evaluated the effect of silicate slag applications on productivity of wheat under sufficient and high N application rates. They applied Si slag at five rates (0, 1, 2, 4.5, and 9 Mg ha^−1^) in combination with two N rates (101 and 145 kg N ha^−1^) and showed that N had a greater impact on wheat productivity than Si application. In addition, their results indicated that Si application effectively raised soil pH, and thus—indirectly—increased availability of several plant-essential nutrients. Martin et al. [29] applied Si as liquid H_2_SiO_4_ to five winter wheat cultivars grown in non-stressed conditions and showed that number of tillers and harvest index were increased due to Si fertilization. Mesoporous Si nanoparticles formed within wheat plant cells were shown to increase wheat seedling growth, seedling photosynthetic activity, and total protein content of seedlings [30]. They also reported that Si application has caused specific changes in the molecular structure of chlorophyll in wheat cells. If Si application can improve plant growth parameters, such as plant height, it would indirectly have a positive effect on crop yield potential. Plant height has been found to be an important indicator of plant growth, biomass production, and yield potential of cereals, including wheat [29].

The majority of previous studies have investigated the effect of Si rate, source, and application time on crop growth separately, and most Si-related work has been focused on assessing the potential of Si to alleviate plant stress. Very few studies have investigated the direct effect of Si rate and application time combinations on irrigated winter wheat growth (plant height), yield, and quality grown in non-stressed environment. The goal of this study was to evaluate the effect of Si rates and application times (at planting and at Feekes 5 growth stage—tillering) on growth, GY, and GP of irrigated winter wheat (v. Stephens) grown in Idaho semi-arid conditions. In addition, to test the hypothesis that Si fertilization may improve uptake of other important plant nutrients, we assessed the effect of Si rate and application time on uptake of several nutrients (phosphorous (P), potassium (K), magnesium (Mg), and calcium (Ca)).

## 2. Results

### 2.1. Plant Height

**Figure 1:**
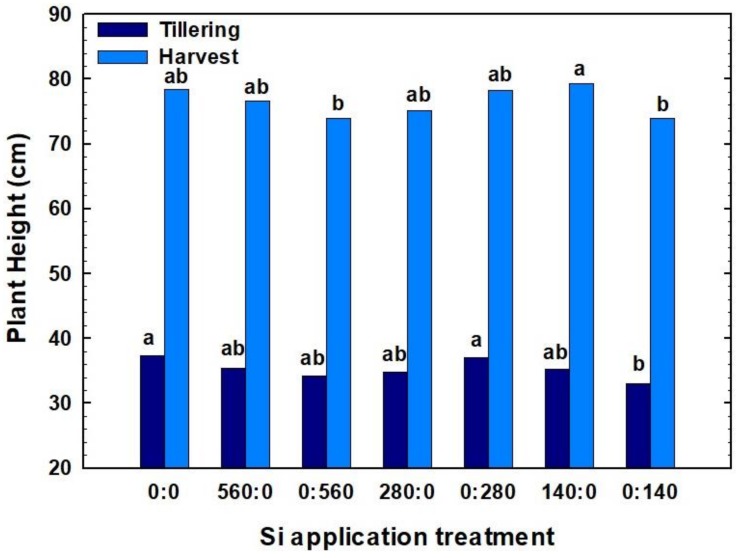
Effect of different silicon (Si) rates and application time on winter wheat height at tillering and before harvest in 2017. Treatments are designated in the format x:y, in which x and y are the fertilizer Si rates in kg Si ha^−1^ applied at planting and at tillering, respectively. Bars within the same year followed by the same letter are not significantly different (*p* > 0.1) based on a Duncan’s multiple range test.

In this study, winter wheat plant heights ranged widely in 2017 (Figure 1). Plant height measured at tillering ranged from 33.0 to 37.3 cm. The tallest plants at this growth stage were observed for the check plot to which no Si was applied. The rate of Si fertilizer applied at planting did not result in significant differences in plant height measured at tillering.

At harvest time, plant height ranged from 74.0 to 79.3 cm. At harvest, the maximum mean plant height was obtained with 140 kg Si ha^−1^ applied at planting. For plant height measured at harvest, there were no consistent trends due to timing of Si fertilization: at planting, fertilization produced taller plants at 140 kg Si ha^−1^ and 560 kg Si ha^−1^ rate, but not at 280 kg Si ha^−1^ rate (Figure 1). 

### 2.2. Content of Si and Other Nutrients in Biomass

**Figure 2:**
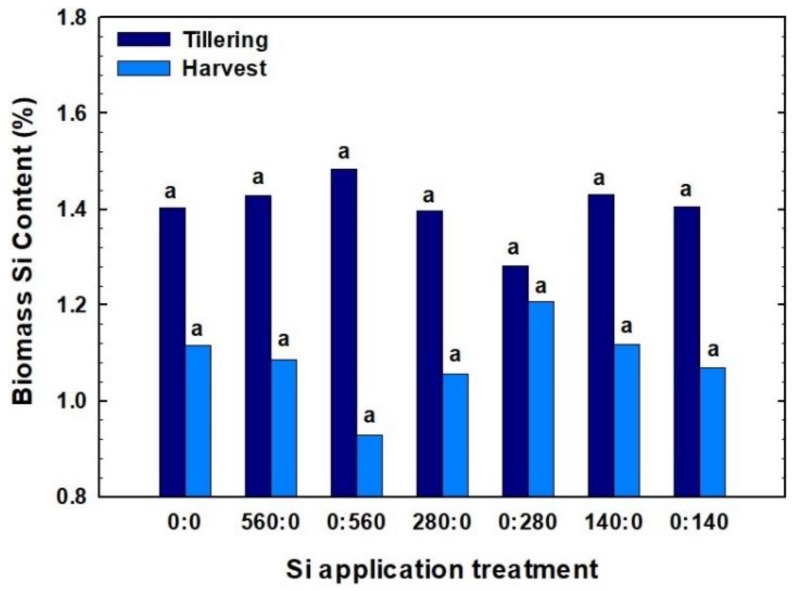
Effect of different Si rates and application time on winter wheat biomass Si content (%) at tillering and before harvest in 2017. Treatments are designated in the format x:y, in which x and y are the fertilizer Si rates in kg Si ha^−1^ applied at planting and at tillering, respectively. Bars within the same year followed by the same letter are not significantly different (*p* > 0.1) based on a Duncan’s multiple range test.

Although the differences were not statistically significant, a range of values for biomass Si content were obtained in 2017 (Figure 2). Biomass Si content measured at tillering ranged from 1.28 to 1.48%. At harvest, biomass Si content ranged from 0.93 to 1.21%, with the highest biomass Si values observed for 560 kg Si ha^−1^ applied at tillering. Biomass Si content decreased consistently from tillering to harvest (Figure 2), likely due to the “dilution effect” as plant size increased and partially due to Si translocation to the grain. 

Typical biomass content of P, K, Mg, and Ca was observed in this study. Phosphorus ranged from 0.3790 to 0.3908%, K ranged from 0.50 to 0.54%, Mg ranged from 0.1193 to 0.1253%, and Ca ranged from 0.045 to 0.050%. Although the differences were not significant, the highest mean P and Ca were observed for the check plot to which no Si was applied. Although numerically higher mean Mg content was obtained at a fertilizer Si rate of 280 kg Si ha^−1^ applied at planting time, it appears that Si application did not improve P, K, Mg, and Ca uptake. For example, while Si rates applied at planting did not significantly affect K content, Si applied at tillering at 140 kg Si ha^−1^ resulted in higher K value, which was identical to that of the no-Si check plot.

### 2.3. Grain Yield and Grain Protein Content

**Figure 3:**
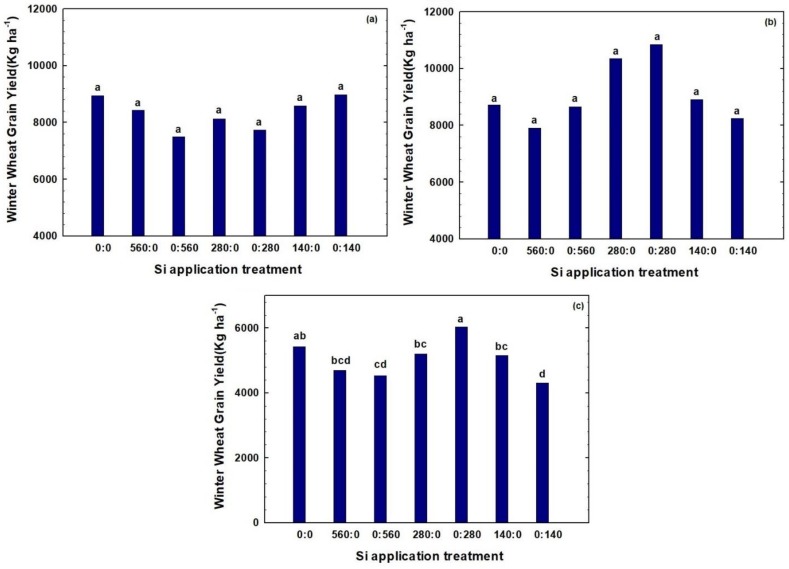
Effect of different Si rates and application time on winter wheat grain yield in (**a**) Field E1 in 2016, (**b**) Field M2 in 2016, and (**c**) Field A3 in 2017. Treatments are designated in the format x:y, in which x and y are the fertilizer Si rates in kg Si ha^−1^ applied at planting and at tillering, respectively. Bars within the same year followed by the same letter are not significantly different (*p* > 0.1) based on a Duncan’s multiple range test.

A wide range of GYs—from 4312 to 10,854 kg ha^−1^—were obtained (Figure 3). In 2016, in field E1, the highest mean GY (8974 kg ha^−1^) was obtained with Si fertilizer rate of 140 kg Si ha^−1^ applied at tillering. In 2016, in field M2, the highest mean GY (10,854 kg ha^−1^) was obtained with Si rate of 280 kg Si ha^−1^ also applied at tillering. Similarly, in 2017, in field A3, the maximum mean GY (6036 kg ha^−1^) was obtained when Si was applied at tillering at the rate of 280 kg Si ha^−1^. Overall, GYs were notably lower in the second year due to harsh weather conditions during the winter months. Though mean temperature and humidity were similar, a cooler wet spring in the second year of study resulted in lower GYs being obtained in 2017, compared to 2016 (Table 1), mainly due to slower plant emergence and establishment. Generally, observed variations in GYs were due to differences in inherent soil fertility and Si rate and application time. When considering all three site-years, the 280 kg Si ha^−1^ produced the best GYs, independent of the application time.

The rate and application times of Si fertilizer did not significantly affect winter wheat GY for either of the two fields in 2016. For field E1, in 2016, application of higher Si fertilizer rates (280 and 560 kg Si ha^−1^), especially at tillering, decreased GY slightly compared to the check plot, but the differences were not statistically significant (Figure 3a). For field M2, in 2016, increasing the rate of Si fertilizer rate at planting to 280 kg Si ha^−1^ resulted in higher wheat GY compared to the check plot, but the differences were not statistically significant. On the other hand, for this site-year, further increasing Si fertilizer rate at planting to 560 kg Si ha^−1^ resulted in relatively lower wheat GYs (Figure 3b). In the second year (field A3), GYs were comparable for all Si applied at planting (Figure 3c). On the other hand, for field A3, the rate of Si fertilizer applied at tillering affected winter wheat GY. While application of Si fertilizer at 140 kg Si ha^−1^ at tillering resulted in significantly lower GY compared to the check plot, increasing the Si fertilizer rate to 280 kg Si ha^−1^ applied at tillering significantly increased GY. This trend did not continue with a further increase in rate of Si applied at tillering—application of 560 kg Si ha^−1^ resulted in relatively lower GYs (Figure 3c).

**Figure 4:**
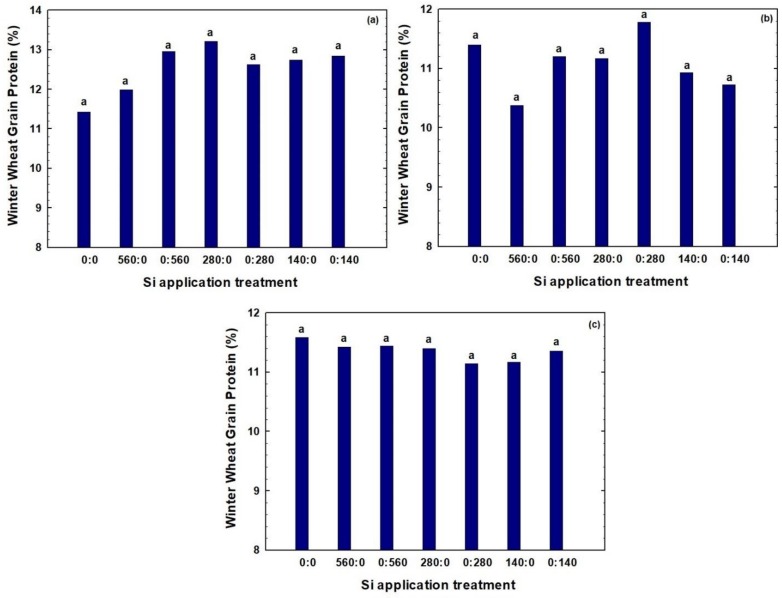
Effect of different Si rates and application time on winter wheat grain protein in (**a**) Field E1 in 2016, (**b**) Field M2 in 2016, and (**c**) Field A3 in 2017. Treatments are designated in the format x:y, in which x and y are the fertilizer Si rates in kg Si ha^−1^ applied at planting and at tillering, respectively. Bars within the same year followed by the same letter are not significantly different (*p* > 0.1) based on a Duncan’s multiple range test.

Grain protein values for soft white winter wheat were typical, ranging from 10.4 to 13.2% (Figure 4). The statistical analysis showed that for all site-years, the rate and application times of Si fertilizer did not significantly affect winter wheat GP. In 2016, for field E1, highest mean GP of 13.2% was achieved at Si fertilizer rate of 280 kg Si ha^−1^ applied at planting time (Figure 4a). For field M2, in 2016, the highest mean GP of 11.8% was observed for plots receiving 280 kg Si ha^−1^ at tillering (Figure 4b). In 2017, in field A3, the maximum mean GP (11.6%) was noted for the non-Si check plot (Figure 4c). 

Although the differences were not statistically significant, and no apparent trends were revealed for GP associated with Si application, in general, Si fertilization slightly increased GP values for field E1. For field A3, GP values decreased slightly with Si application. For field M2, GP values were comparable for all treatments, except for notably lower GP at 560 kg Si ha^−1^ applied at planting. Combining the data for all three site-years, Si application had, indeed, resulted in slightly lower GP values, compared to the non-Si check.

## 3. Discussion

### 3.1. Plant Height

Different studies have shown that Si application positively affects crop growth and yield, as well as plant metabolism and physiology. In terms of plant height, deposition of Si in cell wall can make the leaves and stems more erect and increase plant height [31]. In a pot study by Gong et al. [32], wheat plants grown with Si applied prior to planting had greater plant height compared to plants grown without Si. Similarly, Abro et al. [33] also observed taller plants in rice and wheat, respectively, with Si application. In another study of rice by Gerami et al. [34], plant height was increased following Si application. However, plant height increase was only noted for potassium silicate, and not for sodium silicate, showing that the plant response to Si may be highly dependent on the Si product used as fertilizer source. Ahmed et al. [35] found that Si application increased wheat plant height under both stressed (salinity) and non-stressed conditions; however, the response to Si was greater under water-deficit conditions, compared to non-stressed conditions. The same trend had been noted earlier by Hattori et al. [36], in which Si application in sorghum (*Sorghum bicolor*, L.) had no effect on plant growth under normal moisture conditions. In a field trial, an increase in plant height of rice was observed with the increase of Si application rate; however, the differences were not statistically significant [37]. 

Our findings on the lack of response of wheat plant height to Si fertilization agree with results by Sarto et al. [38] who observed no effect of Si application on wheat plant height or shoot dry matter. One possible explanation for this result could be the source of Si fertilizer. The authors noted that MontanaGrow^TM^ may appeal to organic growers, because it is an Organic Materials Review Institute (OMRI) listed product. In addition, there is very limited published information on MontanaGrow^TM^ showing its efficacy as a fertilizer [39]. Another possibility is that Si fertilization may be beneficial to wheat varieties characterized by taller plants that are susceptible to lodging. Martin et al. [29] noted that differences in characteristics such as capacity for dry matter accumulation and plant height are mainly genetic and linked to other aspects of plant morphology, and thus are not likely to be significantly affected by supplemental fertilization. 

### 3.2. Content of Si and Other Nutrients in Biomass

In our study, the responses of plant Si, P, K, Mg, and Ca status to different Si fertilization treatments were not in line with those obtained by previous studies. Our findings showed that Si application did not improve Si, P, K, Mg, or Ca uptake in wheat, while previous studies have shown that Si application can increase P or Ca uptake in different crops. Silicon application can be very site-specific in terms of affecting various plant parts differently, which could be an explanation for why our findings in this study differed from others. For example, Ma and Takahashi [14] found that rice plants grown with an addition of Si had twice the inorganic P content compared to plants within the non-Si check plots, while Marschner [40] showed that Si has no direct effect on P uptake or translocation to the plant roots. Later, Hanan [41], Liang et al. [42], and Hanafy Ahmed et al. [43] reported that K uptake was significantly increased in plant shoots when Si was added to soil, while in an earlier study Islam et al. [44] had shown that Si generally decreased the K content of rice plants. Neu et al. [45] found that P content of wheat biomass has generally increased with increasing Si rates. They also noted a moderate but significantly positive correlation between Si and P content of wheat biomass. On the other hand, flag leaf P content significantly decreased with increasing Si application rates. 

### 3.3. Grain Yield and Grain Protein Content

Silicon application may enhance crop yield by several indirect actions such as decreased shading due to greater leaf erectness [40]. Ma and Takahashi [46] noted that erectness of leaves as a result of Si fertilization could account for about 10% increase in the photosynthesis, thereby indirectly increasing yield. Similarly, Sorrato et al. [19] showed that Si fertilization increased oat and wheat grain yield by 34.0% and 26.9%, respectively. On the other hand, Segalin et al. [47] found that Si application had no effect on yield or physiological quality of wheat grain. These results agree with those of Marchezan et al. [48] and Freitas et al. [49], who observed no yield increases associated with Si application to rice and corn, respectively. Thus, crops growing under favorable, no-stress conditions, with minimum pest and disease pressure, can achieve optimum grain yields and high seed quality without Si fertilization [50]. In our study, although we did see some positive response of wheat grain yield to Si application—280 kg Si ha^−1^ applied at tillering significantly increased GY in one of three site-years (field A3, in 2017), in most cases no statistically significant differences in GYs associated with Si fertilization were observed in this study.

MontanaGrow^TM^ (0-0-5) by MontanaGrow Inc. (Bonner, MT) used in this study is a Si product sourced from a high-energy amorphous (non-crystalized) volcanic tuff. It contains 76% plant available silicon easily absorbed by plans. The manufacturer lists several potential benefits of the MontanaGrow^TM^ Si, including strengthening of plant roots, stems, and foliage, superior overall crop health with increased stress and disease resistance, higher yields, and increased water use efficiency. However, the application guidelines are not explicit. For wheat, specifically, the potential benefits include boosted GY, grain quality, and increased resistance to lodging [51]. Very little limited published information is available on the use of MontanaGrow^TM^ in wheat or other crops. In an experiment in pumpkin (*Cucurbita pepo* L.), Torlon et al. [39] found that, although MontanaGrow^TM^ is marketed as a Si soil amendment, it did not significantly increase Si concentration in the soil. Furthermore, MontanaGrow^TM^ did little to improve Si uptake in pumpkin and did not enhance crop yield, compared to other Si products evaluated in the study. The authors note that MontanaGrow^TM^ may appeal to organic growers because it is an Organic Materials Review Institute (OMRI) listed product. However, their findings suggested that MontanaGrow^TM^ was not an effective Si fertilizer and did not appear to be a good source of plant available Si. 

## 4. Materials and Methods

### 4.1. Experimental Location

The experimental site is located at the University of Idaho (UI) Southwest Research & Extension Center, Parma, ID, USA (43°48′05.5″ N; 116°56′32″ W, 680 m elevation). The region is characterized as a semi-arid climate with average annual precipitation of approximately 200–300 mm mm and average annual temperature of approximately 10 °C. Mean temperature, total precipitation, and mean humidity for 2016 and 2017 are shown in Table 2. The soil type at this location is Greenleaf-Owyhee silt loam with 0–1% slope. Two fields (E1 and M2) were utilized in the 2015–2016 growing season and one field (A3) was utilized in the 2016–2017 growing season. Soft white winter wheat (var. Stephens) was planted on November 2 in 2015 and October 24 in 2016 at 155 kg ha^−1^ seeding rate using a small plot drill by H&N Equipment (Garden Grove, CA, USA). Following a preplant soil test (Table 3), all plots were treated at seeding with N, P, and K to achieve UI recommended levels for wheat. 

The experimental design was a randomized split plot design with four replications. The main plot treatments were two application times (at planting and at tillering) and subplot treatments were three Si rates (560, 280, and 140 kg Si ha^−1^—corresponding to 100, 50, and 25% of manufacturer recommended rates) (Table 4). Treatment 1 was used as unfertilized check plot to which no Si was applied; it was used to assess wheat response to Si. All plots were treated with MontanaGrow^TM^ Si soil amendment (0-0-5) by MontanaGrow Inc. (Bonner, MT, USA). 

Plots were irrigated using a sprinkler irrigation system. The plots were irrigated weekly 22 April through 8 July (field E1), 5 May through 24 June (M2) in 2016, and 11 May through 6 July (field A3) in 2017 to deliver approximately 40 mm of water for each 8-hour watering period. Plots were mechanically harvested at physiological maturity on 1 August 2016 and 20 July 2017.

### 4.2. Field Data Collection

Plant height (5 randomly selected plants per plot) was measured at tillering, following Si application, and prior to the harvest from each study plot. In addition, whole-plant above ground biomass samples were collected (5 plants per plot) at tillering and at harvest time. Biomass samples were transferred to the lab for total Si content analysis. The biomass samples from harvest time were also analyzed for P, Mg, K, and Ca content. The plant height and biomass nutrient content data was collected for 2017 only. For both years, the winter wheat plots were harvested at maturity, with a Wintersteiger Delta (Salt Lake City, UT, USA) small plot research combine harvester equipped with a mini Grain Analysis Computer (GAC) plus moisture tester Harvest Master Classic Graingage HM800 (Juniper Systems, Inc. Logan, UT, USA) powered by Mirus software (Juniper Systems, Inc. Logan, UT, USA). The harvested wheat grain was dried in the oven at 35 °C for 14 days; then, the dried samples were weighed to determine the accurate by-plot GY, which was adjusted to 12% moisture. Total grain N content was measured using near infrared reflectance spectroscopy (NIR) with a Perten DA 7250 NIR analyzer (Perten Instruments, Inc., Springfield, IL, USA). 

### 4.3. Statistical Analysis

Winter wheat GP content was calculated by multiplying grain total N content by standard 5.7 index. The effect of Si application rate and time on wheat GY, GP, Si, K, P, Mg, and Ca content were evaluated using the analysis of variance (ANOVA). ANOVA was conducted using the PROC GLM procedure in SAS v9.4 (SAS Institute, Inc., Cary, NC, USA) to detect any significant treatment effects. Duncan’s Multiple Range Test was used to separate means at 90% confidence level.

## 5. Conclusions

Silicon has been widely reported to increase the plant growth, biomass, yield, and quality of a wide variety of crops including monocotyledonous crops such as wheat, rice, maize, and barley. These crops have been found to actively take up and accumulate high levels of Si. The observed increases in grain yield, however, may be due not only to the beneficial effects of Si fertilization (such as growth promotion, lodging resistance, and biotic and abiotic stress resistance), but also to some indirect effects like slight pH changes and the uptake of macro- and micronutrients contained in the Si-based fertilizers. In our study, we could not confirm any beneficial effects of Si on plant growth and GY and GP of irrigated winter wheat grown in non-stressed conditions. We acknowledge the fact that our findings may be directly related to the Si fertilizer source used in the study and are planning to further evaluate Si effect on growth and grain production of wheat—grown in non-stressed vs stressed conditions—utilizing several different Si sources and application methods. Furthermore, a greenhouse potted plant study experiment is currently underway to collect additional data on wheat response to Si fertilization.

## Figures and Tables

**Table 1:** Effect of different Si rates and application time on winter wheat biomass P, Mg, K, and Ca content at harvest in 2017.

Si Application Treatment	Nutrient Content (%) of Winter Wheat Biomass			
	P	K	Mg	Ca
0:0	0.3908 a	0.54 a	0.1245 a	0.0500 a
560:0	0.3790 a	0.52 ab	0.1193 a	0.0475 a
0:560	0.3890 a	0.52 ab	0.1238 a	0.0475 a
280:0	0.3855 a	0.51 ab	0.1253 a	0.0450 a
0:280	0.3825 a	0.50 b	0.1235 a	0.0450 a
140:0	0.3800 a	0.51 ab	0.1218 a	0.0475 a
0:140	0.3905 a	0.54 a	0.1223 a	0.0475 a

Means within each column followed by the same letter are not significantly different at *p* < 0.1, as determined by Duncan’s multiple range test.

**Table 2:** Mean temperature, total precipitation, and mean humidity, 2016 and 2017.

	2016	2017
Mean Temperature	10.5 °C	8 °C
Total Precipitation	200 mm	300 mm
Mean Humidity	60%	63%

**Table 3:** Preplant soil test results (0–60 cm).

	2016		2017
	E1	M2	A3
Total N (kg ha^−1^)	67.1	104.4	278.5
P (ppm)	56	26	24
K (ppm)	336	246	344
Organic Matter, %	1.23	3.00	2.47

**Table 4:** Treatment, at planting Si rate, and at tillering Si rate.

Treatment	At Planting Si Rate, kg Si ha^−1^	At Tillering Si Rate, kg Si ha^−1^
1	0	0
2	560	0
3	0	560
4	280	0
5	0	280
6	140	0
7	0	140
